# Anti-Proliferation Potential and Content of Fucoidan Extracted from Sporophyll of New Zealand *Undaria pinnatifida*

**DOI:** 10.3389/fnut.2014.00009

**Published:** 2014-07-10

**Authors:** Wilfred Mak, Sheng Kelvin Wang, Tingting Liu, Nazimah Hamid, Yan Li, Jun Lu, William Lindsey White

**Affiliations:** ^1^School of Applied Sciences, Faculty of Health and Environmental Sciences, Auckland University of Technology, Auckland, New Zealand; ^2^Institute for Applied Ecology New Zealand, Faculty of Health and Environmental Sciences, Auckland University of Technology, Auckland, New Zealand; ^3^School of Interprofessional Health Studies, Faculty of Health and Environmental Sciences, Auckland University of Technology, Auckland, New Zealand; ^4^Institute of Biomedical Technology, Auckland University of Technology, Auckland, New Zealand

**Keywords:** fucoidan, cytotoxicity, fractionation, *Undaria pinnatifida*, sporophyll, extraction

## Abstract

*Undaria pinnatifida* is a species of brown seaweed known to contain rich amounts of fucoidan, a sulfated polysaccharide known to possess various biological activities. We isolated crude fucoidan (F0) from the sporophylls of *U. pinnatifida* grown in the Marlborough Sounds, New Zealand. Sulfate content, uronic acid content, and molecular weight of F0 were 15.02, 1.24, and >150 kDa, respectively. F0 was fractionated to yield three further fractions: F1, F2, and F3. Cytotoxicity of two major fractions was determined by the 3-(4,5-dimethylthiazol-2-yl)-2,5-diphenyltetrazolium bromide (MTT) assay. The algal fucoidans specifically suppressed the proliferation of three cancer cell lines with less cytotoxicity against the normal cells. Selective cytotoxicity could relate to the distinctive structures of each fucoidan fraction. Results from this study provide evidence that fucoidan, especially from *U. pinnatifida* grown in New Zealand, possesses great potential to be used as a functional food to reduce cancer risk or supplement cancer treatment.

## Introduction

Fucoidan is a group of sulfated heteropolysaccharide commonly found in brown seaweeds (1). They are composed primarily of l-fucose residues, and sulfate groups with smaller amounts of d-galactose, d-mannose, d-xylose, d-glucose, uronic acids, and protein (2). Numerous fucoidan types may coexist in the sporophyll of the same algae and their composition varies between species of brown seaweed (3). Since its first isolation from brown seaweed in 1913 (4), fucoidan has been extensively studied. Due to the popularity and increase in demand of pharmaceutical drugs derived from natural sources, it was not until the last decade or so have researchers focused more on the polysaccharides’ broad range of physiological and biological activities (5, 6, 7). These include beneficial cytotoxicity (1), anti-inflammatory (8), antiviral (9), antioxidant (10), and anticoagulant activities (1). As a result, fucoidans have great potential use in the production of new therapeutic agents in the diagnosis and treatment of human diseases. Consequently, research in this area and their cytotoxicity was investigated. Previous studies have also showed that these biological activities were thought to be influenced by their degree of sulfation (11), molecular mass (12), as well as the fucose:sulfate molar ratio (13).

Cancer is a serious disease with complex pathological pathways in the human body. The implementation of natural anticancer agents has been recognized as a possible alternative to conventional chemotherapeutic agents that are associated with minimal survival rates and unpleasant side effects (14). Fucoidan was reported to suppress the growth of cancer cells *in vivo*, and enhances the immune system to subdue the development of tumors (15). Fucoidan was reported to induce a reduction in some types of human cancer cell lines in a dose-dependent manner (16, 17), and clinical trials of fucoidan ingested in human subjects had given promising results (18). However, more research is required in order to promote the use of fucoidan for potential chemotherapeutic treatments. Sulfate content and molecular weight (Mw) of fucoidan polymers have been reported to have a direct relationship to fucoidans’ cytotoxicity and because fucoidan is a polymer mixture, fucoidans extracted from different seaweed or different locations have different bioactivity (19). However, cytotoxicity of lower and higher Mw fucoidans have not been investigated (11, 19).

*Undaria pinnatifida* is a brown seaweed native to Japan, China, and Korea (20) where it is also commercially cultivated for food production (21). It has now spread to over twelve countries including France, Spain, Italy, Argentina, North and South America, Australia, and New Zealand. Their ability to grow in a range of habitats made *U. pinnatifida* an unwanted organism in New Zealand, and was therefore illegal to harvest *U. pinnatifida* commercially (20). Hence structural information on fucoidan from New Zealand *U. pinnatifida* has not been reported. Few studies have been carried out to investigate the anticancer activity of fucoidan extracted from *U. pinnatifida* (11, 16, 19). In particular, knowledge on the bioactivity of fucoidan from New Zealand brown seaweed on human cancer cell lines was absent. Hence the present study was undertaken to first characterize the chemical composition of fucoidan from New Zealand *U. pinnatifida*. Crude fucoidan (F0) was then purified using anion-exchange chromatography to yield three fractions (F1, F2, and F3), and their cytotoxicity were investigated against MCF-7 (breast adenocarcinoma cell line), A-549 (lung carcinoma cell line), WiDr (colon adenocarcinoma cell line), Malme-3M (lung melanoma cell line), HEK-293 (human embryonic kidney cell line), LoVo (human colon adenocarcinoma cell line), HUVEC (Human Umbilical Vein Endothelial Cell line), and HDFb (Human Derma Fibroblast cell line) cells using the 3-(4, 5-dimethylthiazol-2-yl)-2, 5-diphenyl tetrazolium bromide (MTT) cell proliferation assay in comparison with commercial fucoidan (Sigma, USA). The relationship between cytotoxicity and chemical composition was also examined and discussed.

## Materials and Methods

### Materials

*Undaria pinnatifida* was harvested from Port Underwood, New Zealand in September 2011 from two selected mussel farms (farms 106 and 327). The two seaweed samples were independently prepared for extraction. The sporophyll and blade were separated into labeled bags and lyophilized (Christ LOC 1-M Alpha 2-4, Martin Christ, Osterode am Harz, Germany) in bulk within 48 h of frozen storage. The dried sample was milled using a coffee grinder (Breville CG2B), sieved (<0.6 mm) and then stored in PET containers at room temperature before analyses (22). All chemicals and reagents were of analytical grade.

### Extraction of fucoidan

Crude fucoidan was extracted as described previously (13). Each extraction was carried out using sporophylls from separate plants harvested in September, 2011. Four replicate samples of dried algal biomass (15 g) was treated at room temperature for 24 h with a MeOH–CHCl_3_–water mixture (4:2:1) to remove lipids, protein, and colored pigments. The treated algal biomass was filtered through a Whatman’s filter paper (90 mm GF/D), then washed with acetone and dried overnight at room temperature. Treated algae (10 g) were mechanically stirred with 2% aqueous CaCl_2_ (100 mL) at 85°C for 5 h. The extract was centrifuged at 18500 *g* (Eppendorf Centrifuge 5810R V3.1, Eppendorf AG, Hamburg, Germany) and the supernatant was collected. A hexadecyltrimethylammonium bromide (cetavlon) solution (10%, 50 mL) was added to the extract and was left to precipitate at 4°C overnight. The precipitate was centrifuged at 18500 *g*, washed with water and mechanically stirred with 60 mL ethanolic NaI solution (20%) for 72 h. The precipitate was removed by centrifugation, washed with ethanol and lyophilized (Christ LOC 1-M Alpha 2-4, Martin Christ, Osterode am Harz, Germany) to give crude fucoidan (23).

### Determination of sulfate, uronic acid, and protein content

Sulfate content was quantified using the BaCl_2_-gelatin method using K_2_SO_4_ as the standard after hydrolyzing fucoidan (15 mg) in 4 M HCl for 2 h at 100°C (24). Uronic acid and protein content of fucoidan were determined using the carbazole–sulfuric acid–borate reaction using d-glucuronic acid as the standard (25), and the Bradford assay using bovine serum albumin as the standard (26), respectively. Absorbance measurements were recorded using an Ultrospec 2100 UV/visible spectrophotometer. Experiments were performed in triplicates as three independent assays. All yields were calculated from the dried weight of fucoidan and converted to a percentage.

### Fractionation of fucoidan

Crude fucoidan was purified by fractionation using anion-exchange chromatography. Crude fucoidan (2 g) was dissolved in 20 mL of Tris-HCl buffer (0.05 M, pH 7.4) and applied to a column (25 cm × 4 cm) of DEAE–Sephadex A-25 (Pharmacia Ltd.) equilibrated with Tris-HCl buffer connected to a Bio-Rad 2110 fraction collector. The first fraction was eluted with deionized water at a flow rate of 40 drops per tube, followed by NaCl elution at increasing concentrations (1 and 2 M) until the absence of a positive reaction for the presence of sugars in the test tubes when using the phenol–sulfuric acid method according to Dubois et al. (27). Briefly, test tubes containing the eluted samples were transferred (1 mL) into more robust glass test tubes (5 mL). Then 0.05 mL 80% phenol and 2.5 mL concentrated H_2_SO_4_ were added to each test tube and mixed thoroughly. Test tubes were placed on a rack and heated in a 35°C water bath for 20 min. The absorbance was measured at 480 nm (Ultrospec 2100) for any indication of sugars and uronic acids (27). Each carbohydrate-positive fraction was pooled together, dialyzed for 72 h (MWCO 12–14,000) in deionized water with a water change daily and then lyophilized.

### Determination of monosaccharide composition

Monosaccharides were analyzed by gas chromatography of their alditol acetate derivatives (28). Fucoidan (10 mg) was hydrolyzed in 0.5 mL of 2 M trifluoroacetic acid at 121°C in sealed glass tubes flushed with nitrogen for 4 h. After cooling to room temperature, 25 μL of 20 mg/mL allose was added as an internal reference. The hydrolyzate was filtered through a 0.2 μm Phenex-RC syringe filter into clean glass tubes and evaporated to dryness with a gentle stream of filtered air. Milli-Q water (100 μL) was added to the dried hydrolyzates and incubated at 40°C for 90 min after the addition of 20 μL of 15 M ammonia and 1 mL of 0.5 M sodium borohydride in dimethyl-sulfoxide (DMSO). After cooling to room temperature, 1-methylimidazole (200 μL) and acetic anhydride (2 mL) was added to the mixture and incubated for a further 10 min. Milli-Q water (5 mL) was added into each tube to neutralize the excess acetic anhydride and allowed to cool to room temperature. Alditol acetates were extracted by the addition of 1 mL dichloromethane (DCM), and the lower DCM phase was transferred using a Pasteur pipette into a clean glass test tube. This extraction was repeated once more. The combined DCM extracts was washed with 4 mL Milli-Q water and the upper water phase was discarded. The wash was repeated twice more. Alditol acetates were analyzed by GC (GC-2010, Shimadzu) using a ZB-5 capillary column (30 mm × 0.25 mm) coupled to a flame ionization detector. The detector temperature was held at 280°C while the injector temperature was set at 240°C. Nitrogen was used as the carrier gas at a flow rate of 1.5 mL/min and a split ratio of 10. The oven temperature was set at 38°C for 1 min, increased to 200°C at 50°C/min, further increased to 210°C at 2°C/min, and then held for 5 min at the final temperature. Sugar standards used were: l(−)-fucose (Sigma, USA), d(+)-galactose (Serva, Germany), d(+)-xylose (Sigma, USA), d(+)-mannose (Sigma, USA), α-d(+)-glucose (Sigma, USA), and β-d-allose (Sigma, USA). Preparations of sugar standards to their alditol acetate derivatives were identical to that of crude fucoidan. Due to limited resources from farm 106, only *U. pinnatifida* from farm 327 was analyzed for monosaccharide content.

### Determination of average molecular weight

Average Mw of fucoidan from the sporophylls of *U. pinnatifida* was determined by gel permeation chromatography (GPC) using a Sephadex G-100 column (25 cm × 4 cm) equilibrated with phosphate buffer (Bio-Lab Ltd.) (10 mM, pH 7). Crude fucoidan, its fractions (F1, F2, and F3), and commercial fucoidan (10 mg each) were dissolved in 1 mL phosphate buffer and applied to the column at a flow rate of 40 drops per tube. Presence of sugars was detected using the phenol–sulfuric acid method (27). Dextrans (12, 25, 50, 80, 150 KDa) purchased from Sigma were used as standard Mw markers. Blue Dextran 2000 (Pharmacia, Sweden) was used as a void volume marker.

### Cell reducing capacity (cytotoxicity) assay

A-549 cells (lung carcinoma cell line, ATCC CCL-185™), WiDr cells (colon adenocarcinoma cell line, ATCC CCL-218™), MCF-7 cells (breast adenocarcinoma cell line, ATCC HTB-22™), Malme-3M cells (lung melanoma cell line, ATCC HTB-64™), HEK-293 cells (human embryonic kidney cell line, ATCC CRL-1573™), HUVEC cells (human umbilical vein endothelial cell line, ATCC PCS-100-010™), LoVo cells (human colon adenocarcinoma cell line, ATCC CCL-229™), and HDFb cells (human derma fibroblast cell line, Invitrogen NZ Limited) were each cultured in 5 mL of Roswell Park Memorial Institute 1640 (RPMI 1640) complete medium (GIBCO, USA) supplemented with 1% Penicillin–Streptomycin (Invitrogen, USA), 1% l-glutamine (Invitrogen, USA) and 10% fetal bovine serum (Medica Pacifica, USA) at 37°C in a 5% CO_2_ atmosphere.

The cytotoxicity/cell reducing capacity of crude fucoidan (F0) and its fractions (F1 and F3) was determined using the MTT cell proliferation assay (29). Each cell line (A-549, WiDr and MCF-7) was sub-cultured (100 μL) in 96-well plates at a density of 1 × 10^5^ cells per well and allowed to attach to the well bottom for 24 h in 5% CO_2_ at 37°C. Different concentrations (0.2–1.0 mg mL^−1^) of crude fucoidan (F0), its fractions (F1 and F3), and commercial fucoidan (*Fucus vesiculosus*, Sigma, USA) were added into the wells (100 μL) and were incubated for a further 24 h. The supernatant was removed and washed with 150 μL of PBS (pH 7.2) and removed again. The plates were re-incubated for 4 h after the addition of 30 μL of MTT (5 mM, in phosphate buffered saline solution at pH 7.2) reagent and a further 30 min after adding 150 μL of DMSO. Absorbance of each well was measured at 540 nm using a FLUOstar Omega microplate reader (Alphatech, New Zealand). Assays were performed after treatment of cells for 24, 48, and 72 h. The data were used to calculate the cell viability percentage using the equation below. The IC_50_ value was calculated using Prism 5 (GraphPad Software Inc., San Diego, CA, USA).

$$\begin{array}{llll} & \text{Cell Viability }\left(\%\right)\; & & \;\\ & \;=\left(1-\frac{\begin{array}{c} \left(\text{Abs of control}-\text{Abs of blank}\right)\\ \;-\left(\text{Abs of sample}-\text{Abs of blank}\right) \end{array}}{\left(\text{Abs of control}-\text{Abs of blank}\right)}\right)\times 100\; & & \; \end{array}$$

### Statistical analysis

Analysis of variance (ANOVA) was carried out using Minitab^®^ (Version 15) to test for differences between fucoidan fractions. Results of all tests were considered significant if *p* < 0.05. Where significant differences occurred, the Tukey’s Honest Significance Difference (HSD) test was employed to examine where that effect occurred.

## Results

### Crude fucoidan yield and chemical composition

**Figure 1:**
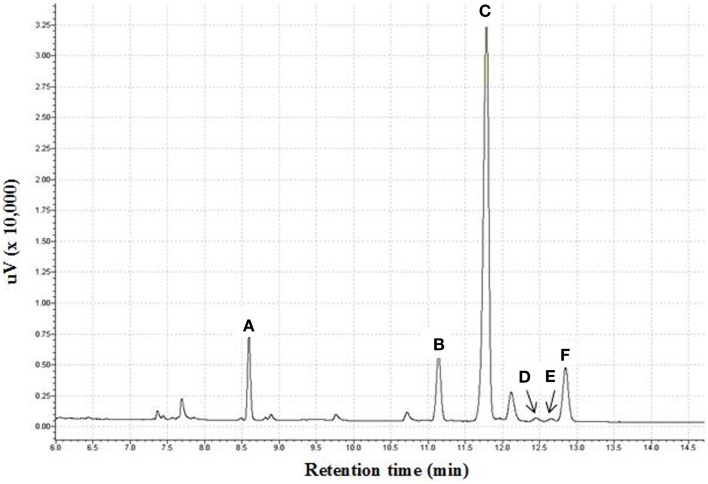
**Gas chromatogram of monosaccharide content of fucoidan obtained from *U. pinnatifida* as alditol acetate derivatives**. **(A)** Fucitol pentaacetate; **(B)** Xylitol pentaacetate; **(C)** Allitol hexaacetate; **(D)** Mannitol hexaacetate; **(E)** Galactitol hexaacetate; **(F)** Glucitol hexaacetate.

Crude fucoidan was extracted from the sporophylls of New Zealand *U. pinnatifida* by CaCl_2_ extraction. Yield of sporophyll-derived fucoidan were 69.98 ± 0.99% and 57.28 ± 6.18% dry weight from farms 327 and 106, respectively. Sporophyll-derived fucoidan contained 7.9 times more fucoidan from site 327 than *U. pinnatifida* harvested in the Kyoungbuk province, Korea while site 106 was 6.5 times more although the extraction method between the two studies was different (19). However, yields of sporophyll-derived crude fucoidan from both sites were slightly lower compared to 71.50% yield previously reported from *Sargassum wightii* collected along the coast of Gulf of Mannar, India (30). As shown in Table 1, crude fucoidan (F0) was shown to be composed primarily of fucose (39.24%), xylose (28.85%), galactose (26.48%), and sulfate (15.02%). The minor components were made up of mannose (5.04%), glucose (0.95%), uronic acid (1.24%), and protein (0.36%). A sample chromatogram of the separate monosaccharides is shown in Figure 1.

**Table 1:** **Chemical composition of crude fucoidan (F0) from *U. pinnatifida*, its purified fractions (F1, F2, and F3) from farm 327 and commercial fucoidan (Sigma)**.

Sample	Uronic acid (%)	Sulphate (%)	Protein (%)	Mw (kDa)	Monosaccharide composition (%)				
					Fuc	Gal	Xyl	Glc	Man
F0	1.24^c^	15.02^d^	0.36^b,c^	>150	39.24^d^	26.48^b^	28.85^a^	0.95^b^	5.04^b^
F1	4.34^a^	6.96^e^	0.86^a^	81	48.51^c^	37.86^a^	3.74^b^	2.91^a^	6.97^a^
F2	0.84^c^	22.78^b^	0.63^b^	22	53.21^b,c^	42.12^a^	1.15^b^	1.28^b^	2.24^c^
F	0.67^c^	25.19^a^	0.11^c^	27	59.71^b^	28.74^b^	1.58^b^	2.77^a^	7.19^a^
Sigma	3.14^b^	17.96^c^	0.41^b,c^	54	87.12^a^	5.69^c^	4.85^b^	0.94^b^	1.39^c^

*^a,b,c,d,e^Column wise values with different superscripts indicate significant differences at *p* < 0.05 by one-way ANOVA followed by the Tukey’s *post hoc* comparison test*.

### Fractionation of crude fucoidan

**Figure 2:**
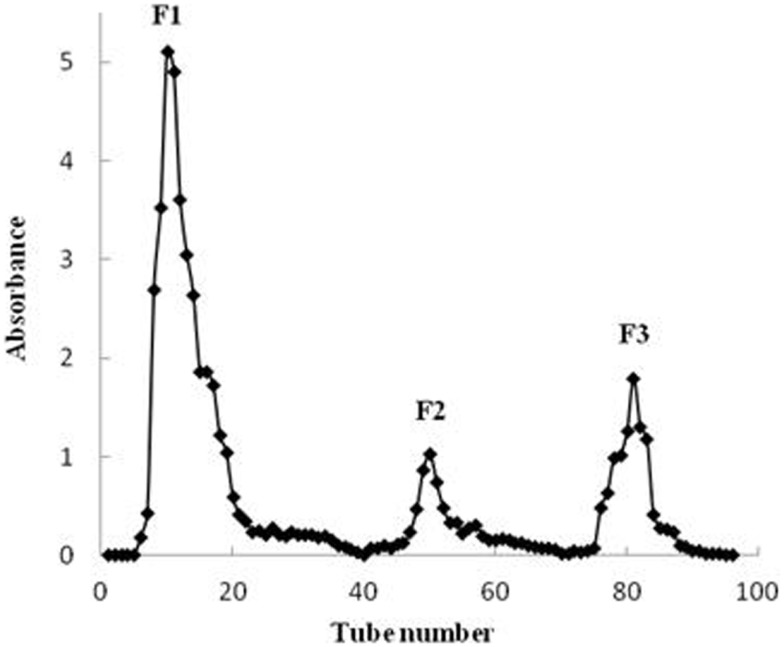
**Chromatogram of purified crude fucoidan extracts obtained from *U. pinnatifida* separated on a DEAE–Sephadex A-25 column**.

Crude fucoidan (F0) was fractionated by DEAE–Sephadex A-25 to yield three fractions, F1, F2, and F3 (Figure 2). No polysaccharide was detected when using 3 M NaCl elution. All fractions contained fucose as the major sugar component followed by galactose, with small amounts of mannose, xylose, and glucose also present. However, the sugar composition varied among the three fractions (Table 1). The fractions were not sulfated xylogalactofucans like crude fucoidan, but were made up of sulfated galactofucans instead. The fraction eluted with a low NaCl concentration was higher in uronic acid and lower in sulfate content. Conversely, the fraction that was eluted with high NaCl concentration was higher in sulfate content and lower in uronic acid. This was similarly encountered with fucoidan from *S. swartzii* (31). The content of fucose increased significantly (*p* < 0.001) from F0 to F3 (Table 1), followed by a significant decrease in xylose content (*p* < 0.001). No noticeable changes between fractions were observed for galactose, mannose, and glucose. Protein content of the different fractions was also similar between F0, F2, and F3, but a significant decrease (*p* = 0.014) in protein content from F1 to F3 fractions indicated less protein contamination when using higher NaCl elution.

### Molecular weight estimation of fucoidan by GPC

The average Mw of crude fucoidan from the sporophylls of New Zealand *U. pinnatifida* was estimated to be over 150 kDa by GPC, after calibration with known standard Mw markers. The mass of crude fucoidan from New Zealand *U. pinnatifida* was higher than crude fucoidan from the sporophylls of the same species harvested from Kijang, Korea that had a Mw of only 38 kDa (32). Conversely, fucoidan extracted from the sporophylls of *U. pinnatifida* grown in Wando, Korea had a much higher average Mw of 2100 kDa (6). The large differences in Mw from the same species were probably attributed to the different fucoidan extraction techniques used that may render fucoidan unstable when heating (33). Variation in Mw of fucoidans from *U. pinnatifida* was also reported by Fitton and Dragar (34). In this study, the Mw of fucoidan fractions eluted with water, 1 and 2 M NaCl were 81, 22, and 27 kDa, respectively (Table 1). The Mw of commercial fucoidan from *F. vesiculosis* purchased from Sigma was estimated to be 54 kDa, which was within the specified range obtained from the Sigma database of 20–200 kDa (35).

### Cell reducing capacity of fucoidan on cancer cell lines

**Figure 3:**
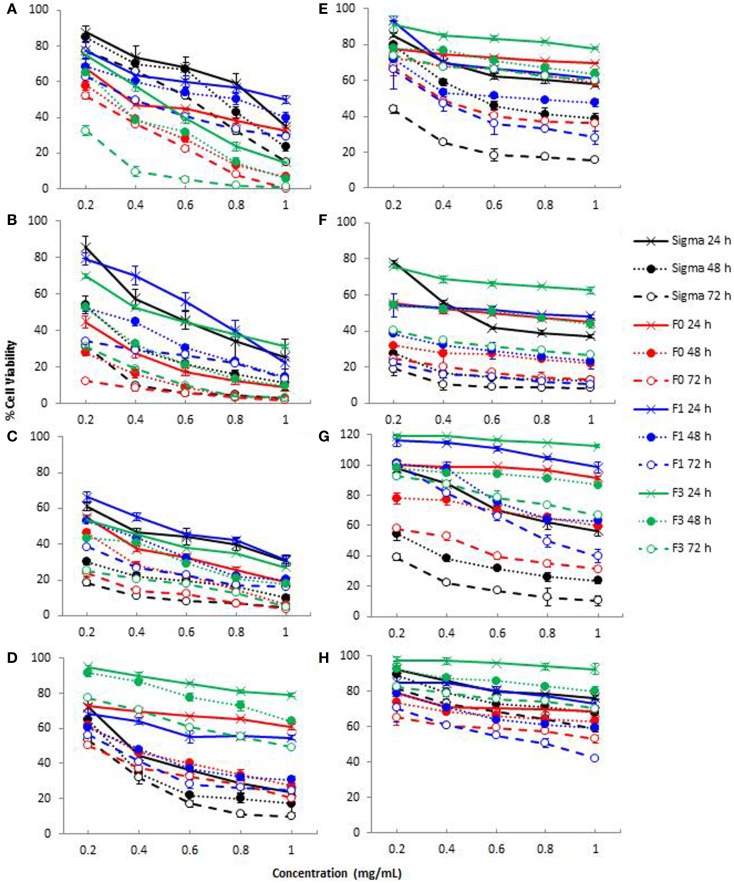
**Cytotoxicity of crude fucoidan (F0), fractionated fucoidan (F1 and F3) and commercial fucoidan (Sigma) against eight cancer or non-cancer cell lines: (A) A-549; (B) MCF-7; (C) WiDr; (D) HDFb; (E) HEK-293; (F) HUVEC; (G) LoVo; and (H) Malme-3M cells after 24, 48, and 72 h incubation**. Results are the means of three independent tests ± SE.

Cell reducing capacity of crude (F0) and purified fucoidan fractions (F1 and F3) toward A-549 (lung carcinoma), WiDr (colon adenocarcinoma), MCF-7 (breast adenocarcinoma), Malme-3M (lung melanoma), and LoVo (human colon adenocarcinoma) cell lines were examined, and compared with commercial fucoidan from Sigma. Each cell line was treated with different concentrations of fucoidan (0.2–1.0 mg/mL) for 24, 48, and 72 h and assessed for cell viability using the MTT cell proliferation assay. This assay was based on the presence of mitochondrial dehydrogenase in viable cells to react with the yellow MTT to form a dark blue–purple formazan complex, which can be solubilized by the addition of DMSO. The amount of viable cells left remaining is directly proportional to the amount of formazan complex produced (36). The cell reducing capacity of fucoidan, expressed as the percentage of viable cells left remaining after fucoidan treatment on MCF-7, A-549, WiDr, Malme-3M, and LoVo cell lines, is shown in Figure 3, while the IC_50_ values (the half minimal inhibitory concentration of a substance) are shown in Table 2. F2 was not included in the cytotoxicity test since this was the smallest of the three purified fractions and was difficult to obtain sufficient amounts required for the MTT assay.

**Table 2:** **Effect of crude fucoidan (F0), its fractions (F1 and F3) and commercial fucoidan (Sigma) toward MCF-7, A-549, WiDr, Malme-3M, LoVo, HDFb, HUVEC, and HEK-293 cell lines at three different incubation times (24, 48, and 72 h)**.

Cell line	Incubation period (h)	Cell type	F0^b^	F1^b^	F3^b^	Sigma^b^
**TUMOR^a^**						
MCF-7	24	Breast adenocarcinoma	0.173	0.651	0.405	0.515
	48		0.024	0.245	0.231	0.221
	72		0.010	0.148	0.110	0.134
A-549	24	Lung carcinoma	0.869	1.018	0.453	0.869
	48		0.278	0.675	0.315	0.744
	72		0.147	0.372	0.242	0.582
WiDr	24	Colon adenocarcinoma	0.244	0.400	0.216	0.321
	48		0.146	0.220	0.173	0.072
	72		0.069	0.121	0.072	0.038
Malme-3M	24	Lung melanoma	>50^c^	>70^c^	>140^c^	>80^c^
	48		>40^c^	>40^c^	>90^c^	>60^c^
	72		>30^c^	>30^c^	>60^c^	>50^c^
LoVo	24	Colon adenocarcinoma	19.090	^d^	^d^	1.548
	48		1.358	2.044	7.596	0.264
	72		0.394	1.051	2.210	0.121
**NON-TUMOR^a^**						
HDFb	24	Derma fibroblast	>40^c^	>30^c^	>90^c^	18.132
	48		18.397	18.436	>70^c^	12.845
	72		13.532	14.542	>40^c^	9.745
HUVEC	24	Umbilical vein	24.828	26.246	>40^c^	26.053
	48		10.108	11.604	24.794	7.142
	72		7.339	6.672	12.854	5.722
HEK-293	24	Embryonic kidney	0.0605	0.057	0.091	>40^c^
	48		0.046	0.030	0.056	28.520
	72		0.022	0.019	0.046	9.523

^a^IC_50_ (mg/mL) was evaluated by the MTT assay, as reported in Section “Cell reducing capacity (cytotoxicity) assay.”

*^b^Results are the mean of three independent experiments*.

*^c^Values are extrapolated*.

*^d^Not determined*.

*Results are expressed as IC_50_ (mg/mL)*.

As shown in Figure 3, treatment of A-549 (A), MCF-7 (B), WiDr (C), LoVo (G), and Malme-3M (H) cell lines with all fucoidan samples showed a dose-dependent and time-dependent relationship at the concentrations (0.2–1.0 mg/mL) tested. The IC_50_ values of crude fucoidan (F0) from this study indicated a higher cytotoxicity than purified fractions (F1 and F3) and commercial fucoidan against both the MCF-7 breast cancer cells and A-549 lung cancer cells after 72 h cultivation (Table 2). At concentrations from 0.2 to 1.0 mg/mL, F0 inhibited the development of cancer cells in a strong dose-dependent relationship and had cell viabilities that ranged from 52.1 to 0.4% against A-549 cells and 12.4–1.7% against MCF-7 cells after 72 h incubation. Moreover, F1 and F3 purified fractions had lower IC_50_ values indicating higher cell reducing capacity than commercial fucoidan when subjected to A-549 cells, while commercial fucoidan showed less cell reducing capacity than F0 and F3 when applied to MCF-7 cells. Conversely, commercial fucoidan exhibited the strongest cell reducing capacity against WiDr colon cancer cells than F0, F1, and F3 with IC_50_ values of 0.069, 0.121, and 0.072 mg/mL, respectively after 72 h incubation. The fact that commercial fucoidan had stronger cell reducing capacity than F0, F1, and F3 although its sulfate content is lower than F3 could be explained by the low Mw and high uronic acid content which both also increase the cell reducing capacity potential of fucoidans (31).

Considerable variations in cell reducing capacity were encountered for both crude fucoidan and fucoidan fractions on different cell lines. Commercial fucoidan showing much higher inhibitory action on WiDr cells than on MCF-7, A-549, Malme-3M, and LoVo cells (*p* < 0.001). Similarly, F1 and F3 fucoidan fractions had lower IC_50_ values for WiDr cells that were associated with a higher inhibitory action than the MCF-7 and A-549 cells. These results reported for the first time that the WiDr colon cancer cells were more susceptible to the inhibitory action of fucoidan compared to the MCF-7 and A-549 cells. A previous study reported that commercial fucoidan from *F. vesiculosis* (Sigma, USA) inhibited the growth of HT-29 colon cancer cells (14), a derivative of the WiDr colon cancer cells (37).

A trend was observed where crude fucoidan exhibited stronger cell reducing capacity than its lower Mw fractions for MCF-7, A-549, and WiDr cell lines. This finding was consistent with the results from a previous study, which reported a stronger inhibitory action of crude fucoidan from *Utricularia aurea* compared to its purified fractions on KB nasopharynx cancer cells (38). Although it has been suggested that fucoidans with a lower Mw and higher sulfate content would provide stronger cell reducing capacity (1, 19), results from this study showed otherwise. This is due to the fact that crude fucoidan in this study, which had the highest Mw of over 150 kDa and a lower sulfate content than the purified fraction F3 (Table 1) exhibited the highest cell reducing capacity. This could be attributed to structural changes during the fractionation process such as depolymerization or by other features in crude fucoidan that may have cell reducing capacity effects (38) that may include the high amount of xylose present in crude fucoidan. Xylose has been used in the past as a component in the development of anticancer drugs (39), and thus there is a reason to believe that xylose content in crude fucoidan may play a vital role in the cell reducing capacity of fucoidans.

### Cell reducing capacity of fucoidan on non-cancer cell lines

Although the cell reducing capacity of fucoidan on certain cancer cells is effective, fucoidan’s selectivity on whether or not to attack non-cancer cells still remains unclear and the mechanisms of how they inhibit tumor growth are still not entirely understood (7). To be able to determine whether the fucoidans in this study had any effect on non-cancer cell lines, assessment of the effects of fucoidan on HUVEC, HDFb, and HEK-293 cell lines were measured using the MTT assay. Cell reducing capacity effects of fucoidan on all three non-cancer cell lines showed a dose-dependent manner, with HUVEC being the most sensitive to crude fucoidan with a reduction of viable cells to 13.0% after 72 h incubation at a concentration of 1 mg/mL crude fucoidan (Figure 3). A drastic reduction in cell viability was encountered for commercial fucoidan from 78.1% cell viability down to 41.7% upon increasing the concentration from 0.2 to 0.6 mg/mL at 24 h incubation. A trend was also noted that F3 had significantly weaker cell reduction rates than F0 and F1 at 0.2–1.0 mg/mL dosage levels for 24 h (*p* < 0.001), but no significant differences were observed between F0 and F1 at the same concentrations and incubation period (*p* < 0.001). Fucoidan also inhibited the growth of HEK-293 and HDFb cells in a dose-dependent fashion. However the extent of inhibition, based on cell viability (Figure 3), was smaller than with the HUVEC cells. The treatment of HEK-293 cells with 1 mg/mL crude fucoidan for 72 h incubation had a 63.7% cell reduction whereas at the same concentration of crude fucoidan and incubation period, HDFb cells had a 79.8% cell reduction, both having a significantly lower cell reduction rate than with WiDr (96.22%), MCF-7 (98.32%), and A-549 (99.96%) cancer cells.

## Discussion

This is the first study to investigate fucoidan’s composition and cytotoxicity extracted from New Zealand *U. pinnatifida*. Since fucoidan is a carbohydrate polymer mixture, fucoidans extracted from different seaweed or from same species but growing at different locations have different bioactivity, including *in vitro* cell reducing capacity in tumor cells (40). Hence, it is important to study fucoidan extracted from *U. pinnatifida* grown in New Zealand to identify whether it has any unique characteristics and biological functions.

The sugar composition and sulfate content indicated that crude fucoidan isolated from the sporophylls of *U. pinnatifida* from New Zealand was made up of sulfated xylogalactofucans. Protein content of New Zealand fucoidan was much lower than in other fucoidans reported (1, 12, 41). This indicated the effectiveness of the extraction technique used in this study to eliminate protein contamination (41). Negatively charged sulfate and uronic acid components of crude fucoidan were more or less lower than fucoidan extracted from *S. swartzii* (31) and *Laminaria japonica* (12). Fucose content in crude fucoidan was somewhat lower than fucoidans extracted from *U. pinnatifida* in Korea (19, 21). However, using different fucoidan extraction methods, galactose and xylose content of fucoidan from New Zealand *U. pinnatifida* were much higher than Korean fucoidan indicating that monosaccharide variations exist within the same species grown in different countries. At present, no reports have reported about xylogalactofucans being extracted from *U. pinnatifida* but there have been reports for other species including *L. angustata* (42), *Sphacelaria indica* (43), and *Spatoglossum schroederi* (44). Fucose content from commercial fucoidan derived from *F. vesiculosus* was significantly higher (*p* < 0.001) than our crude fucoidan. However, xylose, galactose, and mannose content were significantly lower in commercial fucoidan compared to crude fucoidan (Table 1). Variations in fucoidan yield and chemical composition was possibly attributed to the difference in fucoidan extraction techniques, alga harvest season, harvest location, degree of alga maturation, and alga species (3, 32, 45).

A decrease in Mw was observed from F0 to F3 fucoidan fractions as NaCl concentration increased. This change in Mw pattern was also observed with fucoidan extracted from *U. pinnatifida* grown in Peter the Great Bay, using a DEAE–Sephadex A-25 column (3). The decrease in Mw of the fractions was assumed to be caused by depolymerization of fucoidan during the fractionation process (9). In the present work, purified fucoidan fractions from New Zealand *U. pinnatifida* harvested in the Marlborough Sounds, New Zealand was found to be sulfated galactofucans with high Mw. These results also showed that crude fucoidan from New Zealand *U. pinnatifida* were heterogeneous in terms of its sulfate content, uronic acid content, and monosaccharide composition. This is supported by previous studies that have also reported fucoidan from *S. swartzii, Pelvetia canaliculata, F. vesiculosus, S. muticum*, and *L. digitata* as being heterogeneous (31, 46).

The cytotoxicity findings in this study confirmed that crude fucoidan had strong cell reducing capacity, as reported by other studies, using fucoidan from the sporophylls of *U. pinnatifida* (1, 16). The cell reducing capacity of crude fucoidan on MCF-7 cells over 48 h was considerably higher than fucoidan polymers from *A. nodosum* harvested from Ireland, which showed no significant decrease in viable cell numbers over 48 h (47). Similarly, when fucoidan purchased from the NPO Research Institute of Fucoidan, Japan was tested, the number of MCF-7 cells after 48 h incubation increased from approximately 15 × 10^4^ to 18 × 10^4^ cells/well, meaning there was no inhibition of MCF-7 cells. However, a significant decrease in viable cell numbers was only achieved after 96 h incubation period (48). In this study, the degree of inhibition of crude fucoidan on MCF-7 cells was much higher; with only 2.6% viable cells left remaining after 48 h. The results also showed that fucoidan reacted differently to the cell type used, with A-549 cells less sensitive to fucoidans, thereby having a higher IC_50_ value compared to MCF-7 and WiDr cells (Table 2). Fucoidan from the sporophylls of *U. pinnatifida* from South Korea inhibited the growth of A-549 cells from 85.1 to 62.4% at concentrations from 0.2 to 1.0 mg/mL (19). The degree of A-549 inhibition was, however, higher in this study, with 67.5–32.7% inhibition at concentrations from 0.2 to 1.0 mg/mL after 24 h incubation with crude fucoidan. In addition, cell reducing capacity of crude fucoidan from the sporophylls of Korean *U. pinnatifida* against A-549 cells was much lower than crude fucoidan from this study when comparisons were made between the cell viability graphs obtained from their study and ours (16). This confirmed that fucoidan polymers from the same species may result in different cell proliferation capacities that could be due to differences in structural properties and cell type procedures (38).

The results also revealed that fucoidan inhibited dose-dependently of LoVo and Malme-3M cells, although the level of cell reducing capacity was much less than what was encountered with A-549, WiDr, and MCF-7. All fucoidan fractions (F0, F1, and F3) after incubation for 24 h showed significantly less inhibition of LoVo cells when compared to A-549, MCF-7, and WiDr cells at the same incubation period (*p* < 0.001). Commercial fucoidan showed the strongest effect of suppression for LoVo cells at 24, 48, and 72 h incubation than all fucoidan fractions, and showed a lower IC_50_ value than A-549 at 48 and 72 h incubation (Table 2). Both WiDr and LoVo cells are derived from the same tissue region (colon adenocarcinoma) yet the supplementation of both these cells with fucoidan resulted in dissimilar decreases in cell growth capacity (Figure 3). Incubation of WiDr and LoVo cell lines with fucoidan both resulted in dose-dependent decreases in cell growth capacity, with the greatest decreases observed in WiDr cells. It was suggested that the amount of suppression in cell lines of different fucoidans were peculiar and its effects on different kind of cancer cells was different (49). One factor which we believe may have been associated with the differences in inhibiting potential of fucoidan on LoVo and WiDr cells could be the type of grade of the cancer cells, LoVo cells being an advanced bowel cancer type whereas WiDr cells are not. Although no previous studies have indicated this, it is possible that the grade of cell carcinoma could be a factor in fucoidan’s inhibiting potential.

A pattern was also observed for the purified fucoidan fractions, where a decrease in IC_50_ values (Table 2) was evident from fractions F1 to F3, indicating an increase in cell reducing capacity for WiDr, MCF-7, and A-549 cancer cell lines. In addition, it appears that the lower the Mw of the purified fucoidan fraction, the higher the cell reducing capacity. Low Mw and high uronic acid content can increase the cell reducing capacity potential of fucoidans (31). Several studies have confirmed the same effect of having low Mw and high uronic acid content in fucoidans on A-549 and gastric carcinoma cell lines (1, 19). In addition, a significant increase (*p* < 0.001) in sulfate content was encountered from fractions F1 (6.96%) to F3 (25.19%). Studies (11, 19, 50) have indicated that sulfate groups play a major role in the inhibition of cancer cells that may further aid in the suppression of cancer cells. The mechanism by which low Mw-fucoidan polymers result in cytotoxicity has yet to be ascertained. Assumptions have been made that the large Mw-fucoidans have a better chance of having a compact spherical conformation, while low Mw-fucoidans are more likely to exist in a loosed and linear form (1). As anionic sulfate groups may be hidden inside the spherical conformation of a large Mw-fucoidan polymer, this leads to a decrease in the sulfate groups allowed to react with the cancer cells. Therefore, in this study, the observed increase in cell reducing capacity from F1 to F3 may be linked to a combination of a lower Mw and increased sulfate content of the F3 fraction.

Fucoidan from this study had similar effects to commercial fucoidan against non-cancer cell lines, although commercial fucoidan had stronger effects than all fucoidan fractions when administered to HUVEC cells at 1 mg/mL fucoidan for 72 h. Similar studies also reported that fucoidan from *U. pinnatifida* inhibited cell proliferation of HUVEC cultures which in turn may have potential in the prevention of angiogenesis-related diseases (51, 52). From observing Figure 3, commercial fucoidan appeared to have a stronger cell reducing capacity rate than all fucoidan fractions toward HEK-293 and HDFb cells which showed as high as 84.3 and 90.2% inhibition activity at 1 mg/mL dosage level, respectively. Conversely, F3 was the less potent at 24 h incubation for all three normal cell lines, most likely due to its low Mw of only 27 kDa.

In conclusion, crude fucoidan isolated from New Zealand *U. pinnatifida* was a sulfated-xylogalactofucan. It was made up of predominantly fucose, galactose, xylose, and sulfate, which exist along with mannose, glucose, uronic acid, and protein as minor constituents. The results from this study showed that sporophyll-derived fucoidan from New Zealand *U. pinnatifida* had cell reducing capacity effects against MCF-7, A-549, and WiDr cells that was comparable with commercial fucoidan from *F. vesiculosis*. The Mw and degree of sulfation appeared to be related to the cytotoxicity of purified fucoidan fractions. Fucoidan content is high in the sporophyll of New Zealand *U. pinnatifida* which is normally regarded as waste and would therefore be beneficial to derive fucoidan from the sporophyll of this alga. Since fucoidan has the potential to reducing cancer cell growth, the sporophyll of New Zealand *U. pinnatifida* can be a good resource for manufacturing health products or produce food for health. The results have also shown that extracted fucoidan exerts cell reducing capacity effects in a variety of cancer cells. Even though it has toxicity at high concentration, it exhibits anti-proliferation effect in cancer cells at concentration lower than the normal cell toxic level. We believe it is possible to be used to combine with other chemotherapeutic agents to treat cancer. Therefore, further studies on fucoidan extracted from New Zealand *U. pinnatifida* are warranted, possibly in animal or clinical trials.

## Conflict of Interest Statement

The authors declare that the research was conducted in the absence of any commercial or financial relationships that could be construed as a potential conflict of interest.
